# Association between dietary iron intake and pelvic inflammatory disease in women from the US: Findings from the 2013 to 2020 NHANES dataset

**DOI:** 10.1097/MD.0000000000048490

**Published:** 2026-05-08

**Authors:** Xiaoshi Wang, Jia Ye, Xiaoteng Chen, Qingsong Zhang, Jinwei Zhang

**Affiliations:** a Wuxi Medical Center, Nanjing Medical University, Wuxi, Jiangsu, China; b Wuxi People’s Hospital, Nanjing Medical University, Wuxi, Jiangsu, China.

**Keywords:** dietary iron intake, dietary trace elements, national health and nutrition examination surveys (NHANES), pelvic inflammatory disease (PID), smoothed curve fitting, threshold analysis

## Abstract

Pelvic inflammatory disease (PID), a prevalent gynecological ailment compromising women’s health, demonstrates established risk mitigation from dietary trace minerals. However, iron’s role in PID pathogenesis remains unestablished. The National Health and Nutrition Examination Survey collected data on dietary iron intake and PID through dietary intake questionnaires and reproductive health questionnaires. We utilized data from 2013 to 2020 and employed multiple logistic regression to explore this relationship. This was further enhanced by smooth curve fitting and threshold analysis to determine linear and nonlinear relationships. Subgroup analyses based on various demographic characteristics were also executed. Our cohort comprised 5034 women between the ages of 20 and 59. Findings from the multivariate logistic regression suggested a protective effect of higher iron consumption against PID. Subgroup analyses underscored a marked reduction in PID risk among women who were cohabiting, diabetic, and nonsmokers. The application of smoothed curve fitting illustrated a U-shaped curve describing the relationship between iron intake and PID risk. Threshold analysis indicated a decrease in PID risk by 3% per unit increase in iron intake below 27 mg/day. Additional univariate and multivariate logistic regression analyses of other common dietary trace elements and vitamins revealed no significant independent association with PID risk after full covariate adjustment, which highlighted the specific protective role of dietary iron in PID among dietary trace elements. Our analysis revealed a significant inverse association between iron intake and PID development, particularly when iron consumption was below 27 mg/day. These findings emphasize the crucial role of trace minerals in enhancing gynecological health and propose that augmenting dietary iron could be a strategic approach to prevent PID.

## 1. Introduction

Pelvic inflammatory disease (PID) represents a spectrum of common gynecologic inflammatory disorders such as endometritis, salpingitis, tubo-ovarian abscess, and pelvic peritonitis.^[1]^ Predominantly affecting sexually active women, these disorders are characterized by infection and inflammation in the upper genital tract, frequently caused by pathogens like Neisseria gonorrhoeae and Chlamydia trachomatis.^[2]^ Although treatable in many cases, immediate medical attention is imperative. Delayed treatment can result in grave outcomes including escalated risks of ectopic pregnancy, secondary infertility, chronic pelvic pain, and adverse pregnancy results.^[3]^ With annual incidences reported between 500,000 and 1,000,000 in the United States,^[4]^ and treatment costs averaging $3025,^[5]^ PID imposes a substantial strain on healthcare resources and public health systems. It is imperative, therefore, to further investigate PID risk factors and implement early interventions based on sound medical evidence.

Dietary trace minerals, though minor constituents of the diet, play pivotal roles in metabolic functions. Key trace minerals include copper, iron, selenium, and zinc.^[6]^ Iron is especially critical for health, contributing to protein synthesis, oxygen transport, and immune function.^[7]^ Deficiencies in iron can lead to various health issues, including iron-deficiency anemia, atrophic ligamentitis, and restless leg syndrome, whereas excess iron intake has been linked to biotoxic effects such as oxidative DNA damage.^[8]^ Recent research has associated high iron and zinc levels with negative effects on conditions like fibromyalgia, which is known for widespread musculoskeletal pain, and may also influence other chronic symptoms including anxiety and depression. Elevated iron intake has also been associated with a higher diabetes risk.^[9]^ However, the specific role of iron in PID remains to be elucidated.

Consequently, this study leverages National Health and Nutrition Examination Survey (NHANES) data from 2013 to 2020 to explore the potential link between dietary iron intake and PID, aiming to furnish insights that could inform preventive and therapeutic strategies against PID.

## 2. Methods

### 2.1. Data source and participants

The NHANES data is a comprehensive, stratified, multistage interview survey conducted throughout the U.S. All participants undergo an initial demographic interview at home, followed by a secondary interview or various health screenings in a mobile examination center for various health screenings.

**Figure 1.**
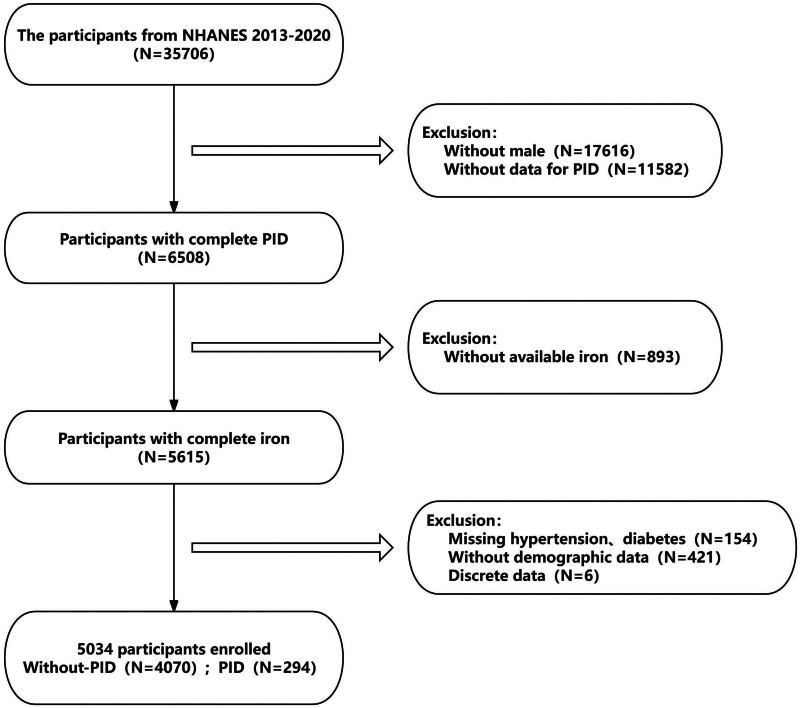
Flowchart of participant selection from NHANES 2013 to 2020. N = number of participants, PID = pelvic inflammatory disease.

This was a cross-sectional study with participant data from 4 NHANES survey cycles (2013–2014, 2015–2016, 2017–2018, and 2019–2020) totaling 35,706 participants, of which 18,090 were female, 165 in a state of pregnancy, were initially selected. After excluding participants with missing data on PID, 6508 participants remained, followed by the exclusion of those with incomplete dietary intake data, 5615 participants remained, 581 of whom were excluded due to missing data on body mass index (BMI), marital status, hypertension, diabetes mellitus, or abnormal dietary intake. Finally, a total of 5034 women aged 20 to 59 years were included, of whom 294 had PID (Fig. 1).We had planned to exclude all pregnant women from all study cycles due to the fact that “pregnancy” can cause abnormalities in iron intake and metabolism in women (Reproductive Health-RHD143-Are you pregnant now?). Coincidentally, when we initially excluded a large number of participants with missing data, we found that those who were pregnant were also excluded.

The Ethical Review Board of the National Center for Health Statistics granted approval for this study, and all participants consented in writing. No additional external ethical clearances were deemed necessary (continuations of protocols #2011-17 and #2018-01).

### 2.2. Measurement of PID

For PID diagnosis, the reproductive health questionnaire from the Questionnaire data section on the official website should be consulted, specifically question RHQ078 (Has the subject ever received treatment for an infection in the fallopian tubes, uterus, or ovaries, also recognized as pelvic infection, PID). A “Yes” response indicates a PID diagnosis, while “No” suggests the absence of PID.^[10]^

### 2.3. Measurement of dietary iron intake

The NHANES dietary intake survey utilizes the 24-hour recall questionnaire “What We Eat In America,” conducted by the U.S. Department of Agriculture in partnership with the U.S. Department of Health and Human Services. The questionnaire compiled detailed data on all food and beverages consumed in the past 24 hours (midnight to midnight), assessed nutritional types and amounts, and calculated intakes of energy, nutrients, and other food components.^[11]^ The dietary supplement use component is conducted after the 24-hour dietary recall, and all NHANES participants who have been interviewed for the 24-hour dietary recall answer questions about dietary supplement and over the counter antacid use. As a result, NHANES is provided with information on the use of all vitamins, minerals, herbs, and other dietary supplements, as well as over the counter antacids, including the name of the supplement or antacid and the amount taken. In addition, participants also provided information on whether they are currently following any type of diet aimed at weight loss or for other health-related purposes (such as a vegan diet). The variable DRQSDIET identifies whether participants are adhering to a special diet. These variables can be found in the “Total Nutrient Intake file.”

We included all data obtained from both questionnaires, calculated the mean of iron intake, and used data from the first day if data from the second interview were missing. In addition, we refer to the Recommended Dietary Allowance (RDA) for women (iron 18 mg/day).^[12]^ Participants whose intake exceeded 10 times the RDA were defined as discrete value and excluded.

### 2.4. Assessment of covariates

Leveraging insights from prior research,^[13,14]^ our study incorporated demographic variables (age, race, education, marital status), socioeconomic indicators (poverty income ratio [PIR]), health and lifestyle metrics (BMI, smoking status, diabetes, and hypertension), and reproductive health measures (menstrual regularity) as covariates. These data, including demographic variables, PIR, and BMI, are directly accessible via the NHANES website.^[15,16]^ The NHANES Cigarette Use Questionnaire detailed participants’ smoking behaviors^[17]^; those answering “Yes” to “SMQ020 - Smoked at least 100 cigarettes in life” were identified as smokers. Information regarding the regularity of menstruation was derived from the Reproductive Health Questionnaire, excluding those who were pregnant or affected by surgical interventions.

We used a multiple data analysis model for the diagnosis of hypertension and diabetes. Participants were diagnosed as having diabetes if they answered “Yes” to “DIQ010 - Doctor told you have diabetes” on the diabetes questionnaire; were using hypoglycemic/insulin medication; had a glycosylated hemoglobin test > 6.5% or had a fasting plasma glucose test > 7.0 mmol/L. Participants who met any of these criteria were diagnosed with diabetes. In the same way, participants who answered “Yes” to “BPQ020 - Ever told you had high blood pressure (BP)” on the Hypertension Questionnaire; using BP-lowering medication;the average of 3 consecutive measurements of systolic BP ≥ 140 mm Hg or diastolic BP ≥ 90 mm Hg (if data from 1 measurement were missing, the data from the other 2 measurements would be used). Those who fulfilled any of these conditions were diagnosed with hypertension.^[18]^ In addition, we classified positive participants’ glycemic and BP control based on glycated hemoglobin (7% cutoff) and the mean of BP measurements (140/90 mm Hg cutoff).

### 2.5. Statistical analysis

Statistical analyses were conducted using R (v4.2.0; https://cran.r-project.org/), R Studio (v1.3.1093; https://cran.r-project.org/), and EmpowerStats (v4.1; https://www.empowerstats.net/cn/), with dietary weights applied to enhance the precision and accuracy of the study, following NHANES sampling guidelines.^[19]^ Data were reported as mean and standard deviation.

Multivariate logistic regression analyses were performed to elucidate the relationship between dietary iron intake and PID. Initially, iron intake was treated as a continuous variable, with smoothed curve fitting applied to delineate the nonlinear dose-response curve. Iron intake was then segmented into tertiles for deeper analysis: T1 (< 31.5 mg/day); T2 (31.5–63.1 mg/day); T3 (63.1–94.6 mg/day), with T1 as the reference group. The analysis was progressively refined through different models (Models I–III): Model I incorporated no adjustments; Model II adjusted for age and ethnicity; and Model III expanded adjustments to include educational level, marital status, PIR, BMI, menstrual regularity, smoking status, and presence of diabetes and hypertension.^[20]^ Additional subgroup analyses and interaction tests were employed to validate the consistency of these associations across diverse demographic profiles.

Based on the same cohort and covariate adjustment strategy, we also performed univariate and multivariate logistic regression analyses on other common dietary trace elements and vitamins to explore their potential associations with PID.

The relationship between iron intake and PID was quantified using odds ratios (ORs). Statistical significance was established at *P* < .05.

## 3. Result

### 3.1. Population description

After screening, 5034 women were included in this study with a mean age of 39.93 years. The highest percentage of Non-Hispanic White of all races; mostly cohabiting with a partner; and most with higher education. A total of 294 participants diagnosed with PID had lower dietary iron intake (11.75 mg/day); were older; had a higher BMI; had a lower household income; more likely to suffer from hypertension; were more likely to be smokers and generally have irregular menstrual cycles (all *P* < .01) (Table 1).

**Table 1:** Characteristics of all participants.

Characteristics	Overall	Without PID	PID	Standardize diff.	*P* value
	(N = 5034)	(N = 4740)	(N = 294)		
Iron (mg/d)	12.09 ± 6.92	12.11 ± 6.81	11.75 ± 8.50	0.05 (−0.07–0.16)	.002
Age (yr)	39.93 ± 11.55	39.74 ± 11.58	42.94 ± 10.70	0.29 (0.17–0.41)	< .001
BMI (kg/m^2^)	30.52 ± 8.56	30.42 ± 8.54	32.15 ± 8.68	0.20 (0.08–0.32)	< .001
Race (n,%)				0.33 (0.21–0.45)	< .001
Mexican American	755 (15)	733 (15.46)	22 (7.48)		
Non-Hispanic White	1747 (34.7)	1640 (34.60)	107 (36.39)		
Non-Hispanic Black	1242 (24.67)	1139 (24.03)	103 (35.03)		
Other Races	1290 (25.63)	1228 (25.91)	62 (21.09)		
Education (n, %)				0.05 (−0.07–0.17)	.724
Below high school	253 (5.03)	236 (4.98)	17 (5.78)		
High school	524 (10.41)	491 (10.36)	33 (11.22)		
Above high school	4257 (84.56)	4013 (84.66)	244 (82.99)		
Marital (n, %)				0.14 (0.02–0.25)	.078
Living together	4731 (93.98)	4458 (94.05)	273 (92.86)		
Living alone	303 (6.02)	282 (5.95)	21 (7.14)		
PIR (n, %)				0.19 (0.07–0.31)	.002
Low (< 3.0)	2579 (51.24)	2402 (50.68)	177 (60.20)		
High (≥ 3.0)	2455 (48.76)	2338 (49.32)	117 (39.80)		
Hypertension (n, %)				0.35 (0.23–0.46)	< .001
Yes	1390 (27.61)	1264 (26.67)	126 (42.86)		
No	3644 (72.39)	3476 (73.33)	168 (57.14)		
Diabetes (n, %)				0.07 (−0.05–0.19)	.234
Yes	577 (11.46)	537 (11.33)	40 (13.61)		
No	4457 (88.54)	4203 (88.67)	254 (86.39)		
Smoke (n, %)				0.52 (0.40–0.64)	< .001
Yes	1638 (32.54)	1473 (31.08)	165 (56.12)		
No	3396 (67.46)	3267 (68.92)	129 (43.88)		
Regular period (n, %)				0.25 (0.13–0.36)	< .001
Yes	3402 (67.58)	3236 (68.27)	166 (56.46)		
No	1632 (32.43)	1504 (31.73)	128 (43.54)		

The format of mean ± SE is used to present continuous variables, whereas counts and percentages are used to present categorical variables. Categorical and continuous characteristics were analyzed using the chi-square tests and *t* tests, respectively.

BMI = body mass index, d = day, kg = kilogram, m = metre, mg = milligram, N/n = number of participants, PID = pelvic inflammatory disease, PIR = poverty income ratio, SE = standard error.

### 3.2. The associations between dietary iron intake and PID

Our analysis involved multivariate logistic regression, considering dietary iron intake as both a continuous and a categorical variable segmented into tertiles. Although treating iron intake as a continuous variable did not reveal any significant associations, a pronounced decrease in PID risk was identified in the highest tertile group (T3, 63.1–94.6 mg/day) compared to the lowest tertile group (T1, < 31.5 mg/d). The ORs for PID in the T3 participants compared to the T1 participants were 0.61 (*P* = .0014) without any adjustment for confounders, while the ORs for PID after adjusting for age and race were 0.65 (*P* = .0058). In the fully adjusted models (Models III), the adjusted ORs were 0.70 (95% confidence interval [CI]: 0.52–0.95, *P* = .0231) for the T3 participants as compared to the T1 participants.The statistically significant result indicating that higher dietary iron intake was protective against the development of PID (Table 2).

**Table 2:** Multivariable logistic regression to assess the association of iron intake with PID.

	Model I		Model II		Model III	
	OR (95% Cl)	*P* Value	OR (95% Cl)	*P* Value	OR (95% Cl)	*P* Value
Iron	0.99 (0.97–1.01)	.3865	1.00 (0.98–1.01)	.6042	1.00 (0.98–1.02)	.9551
Categories (mg/d)						
T1 (0-31.5)	Ref		Ref		Ref	
T2 (31.5–63.1)	0.87 (0.66–1.15)	.3291	0.88 (0.67–1.16)	.3752	0.96 (0.73–1.27)	.7706
T3 (63.1–94.6)	0.61 (0.46–0.83)	.0014	0.65 (0.48–0.88)	.0058	0.70 (0.52–0.95)	.0231

Model I: no adjustment. Model II: adjust for age; race.

* Model III: adjust for all covariates (age; race; education; BMI; PIR; marital; regular period; hypertension; diabetes; smoke).

BMI = body mass index, CI = confidence interval, d = day, mg = milligram, OR = odds ratio, PID = pelvic inflammatory disease, PIR = poverty income ratio.

### 3.3. The detection of nonlinear relationships

**Figure 2.**
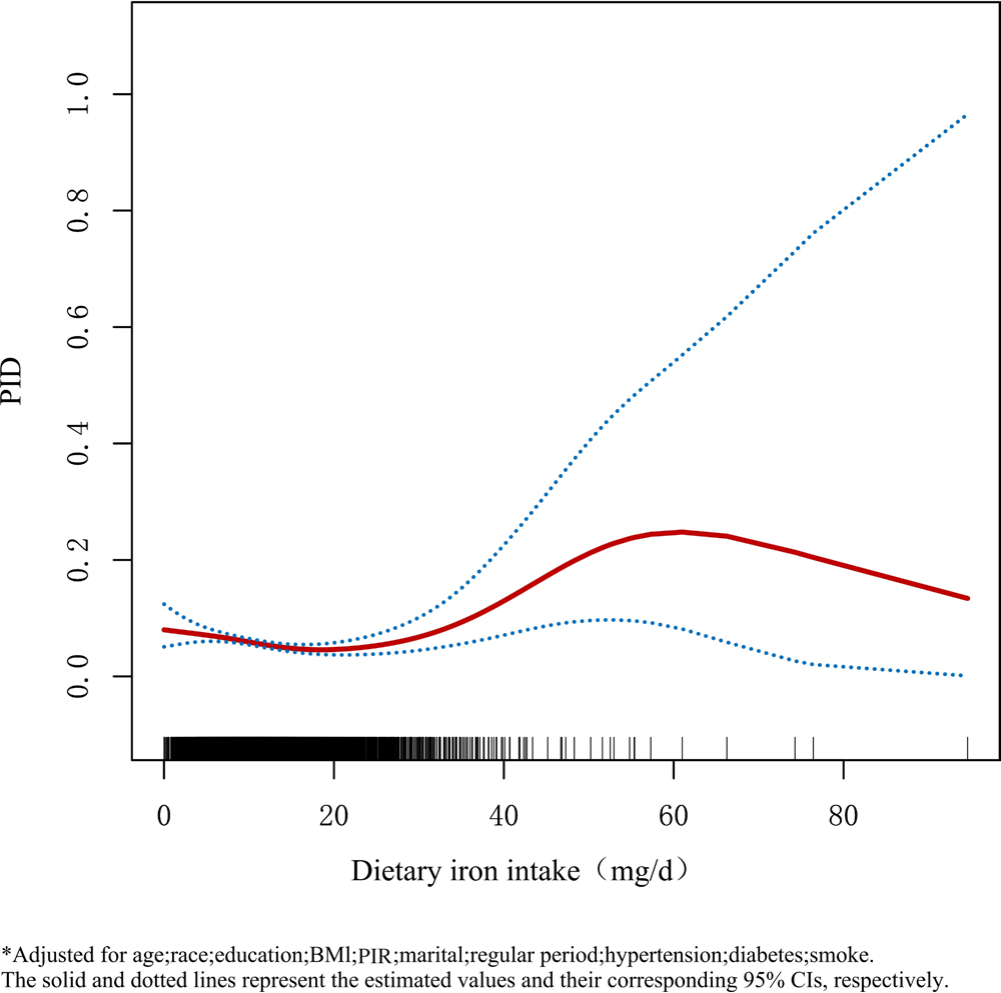
Smoothed curve fitting. BMI = body mass index, CI = confidence interval, PID = pelvic inflammatory disease, PIR = poverty income ratio.

We adjusted for all covariates and found that the relationship between dietary iron intake and PID resembled a U-shaped curve by generalized additive models and smoothed curve fitting analysis(Fig. 2),which means there are nonlinear negative and positive correlations at different intake levels. We performed Threshold analysis to further understand the relationship. As shown in Table 3, we calculated the threshold effect of the relationship between the 2 inflection points of dietary iron intake of 18 and 36 mg/day and 27 and 54 mg/day in relation to the risk of PID by multiplying the dietary iron intake by the RDA recommendation. When 27 and 54 mg/day were used as inflection points, dietary iron intake below 27 mg/day decreased the PID-adjusted ORs by 3% (95% CI: 0.95–0.99) for every 1-unit increase in intake, and the PID-adjusted ORs increased by 11% (95% CI: 1.05–1.18) for every 1-unit increase in intake at dietary iron intake between 27–54 mg/day. The *P* values were all < .05 and the log-likelihood ratio was < .001 making the results statistically significant.Whereas,the results were not statistically significant when dietary iron intake was > 54 mg/day (*P* = .3642).

**Table 3:** Threshold effect analysis of dietary iron intake on PID.

	Adjusted OR (95% Cl)	*P* value
Fitting by the standard linear model	1.00 (0.98–1.02)	.9551
Fitting by the 2-piecewise linear model		
Inflection point (mg/d)	18–36	
Iron intake < 18mg/d	0.95 (0.93–0.98)	.0022
Iron intake 18–36mg/d	1.05 (1.00–1.11)	.0675
Iron intake > 36mg/d	1.02 (0.96–1.09)	.5011
*P* for Log-likelihood ratio		< .001
Inflection point (mg/d)	27–54	
Iron intake < 27mg/d	0.97 (0.95–0.99)	.0128
Iron intake 27–54mg/d	1.11 (1.05–1.18)	.0003
Iron intake > 54mg/d	0.85 (0.60–1.21)	.3642
*P* for Log-likelihood ratio		< .001

* Adjust for all covariates (age; race; education; BMl; PIR; marital; regular period; hypertension; diabetes; smoke).

BMI = body mass index, CI = confidence interval, d = day, mg = milligram, OR = odds ratio, PID = pelvic inflammatory disease, PIR = poverty income ratio.

### 3.4. Subgroup analyses

**Figure 3.**
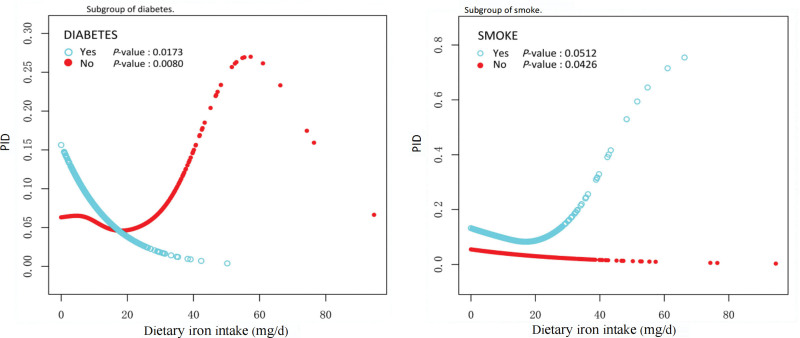
Subgroup of smoke & diabetes. PID = pelvic inflammatory disease.

In Table 4, stratified analyses within the fully adjusted model demonstrated significant negative associations between dietary iron intake and PID in subgroups comprising individuals who were cohabiting, diagnosed with diabetes, and nonsmokers (*P* < .05). In contrast, a positive association was observed in the subgroup of women who lived alone (*P* = .0469). To analyze the effect of interactions in significant subgroups, we built generalize additive models and smoothed curve fitting based on their stratification for further analysis. As shown in Figure 3, stratified analyses of the Smoke subgroup showed a similar linear negative correlation between PID and iron intake in the never smokers (*P* = .0426) and an L-shaped negative correlation between PID and iron intake in the diabetes subgroup in the diabetic group (*P* = .0173).

**Table 4:**
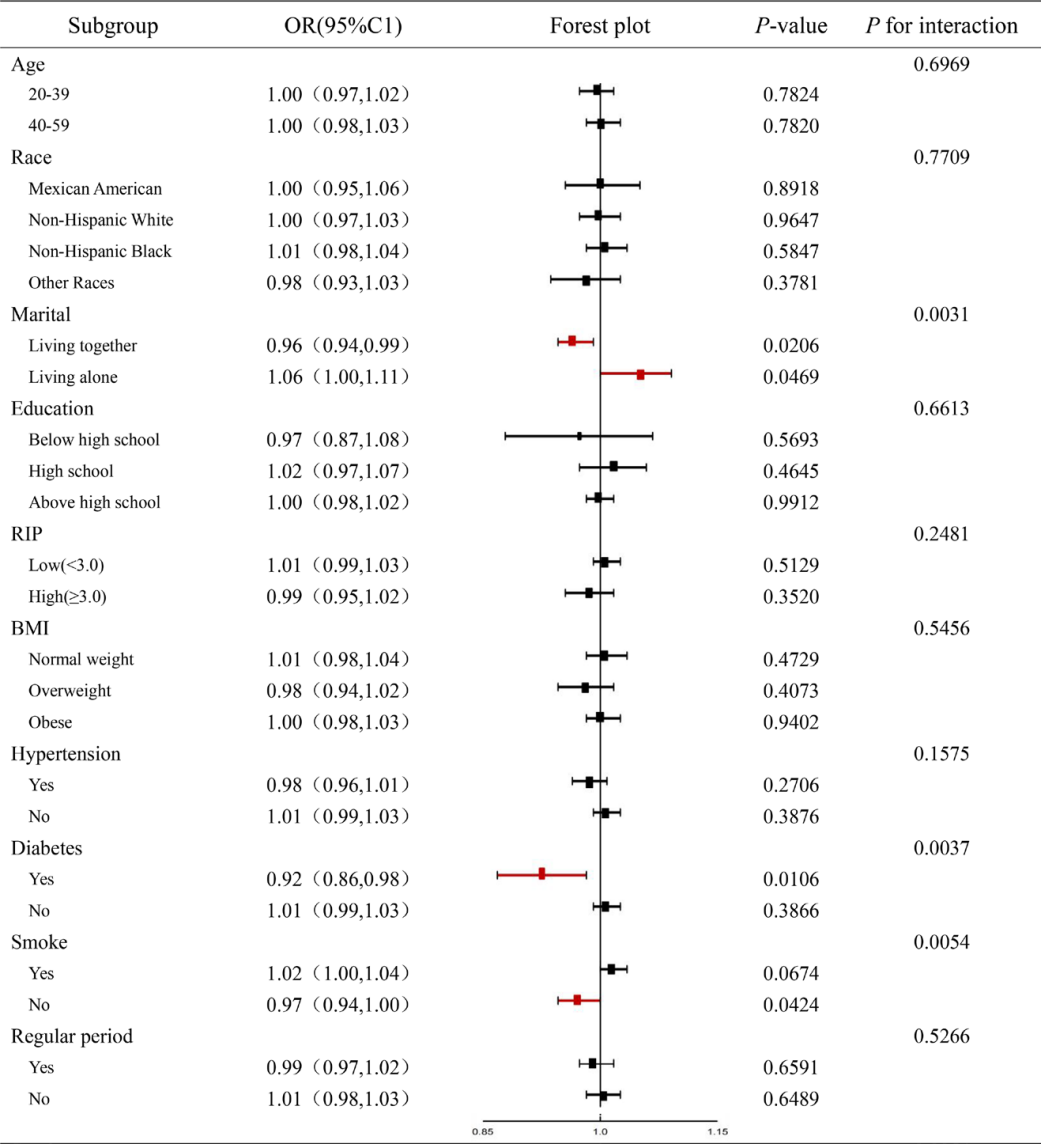
Subgroup analysis between dietary iron intake and the risk for PID.

### 3.5. Association between other dietary trace elements and PID

To provide evidence for formulating a nutritional regime synergistic with the therapeutic scheme for PID, we conducted univariate and multivariate logistic regression analyses for other common dietary trace elements (Ca, Cu, Mg, P, Se, Zn, Na, K) and vitamins (VB1, VB2, VB6, VB12, VC, VD, VK) based on the same study cohort and the fully adjusted covariate model (Model III) consistent with the iron intake analysis.

Univariate analysis showed that Ca, Mg, P, Zn, and Na were significantly associated with the risk of PID (all *P* < .05), while other elements and vitamins had no statistically significant correlations. However, after full adjustment for all confounding factors (age, race, education, BMI, PIR, marital status, etc) in the multivariate logistic regression model, all the above dietary trace elements and vitamins exhibited no significant independent association with PID risk (all *P* > .05). Notably, Zn presented a marginal protective trend in the multivariate model (OR = 0.97, 95% CI: 0.93–1.02, *P* = .222) but did not reach statistical significance. (Table 5)

**Table 5:** Univariate and multivariate analysis of dietary trace elements with PID.

Characteristic	Univariable			Multivariable		
	OR	95% CI	*P value*	OR	95% CI	*P value*
Ca	1.00	1.00, 1.00	.021	1.00	1.00, 1.00	.854
Cu	0.93	0.77, 1.12	.401	0.94	0.75, 1.18	.597
Mg	1.00	1.00, 1.00	.003	1.00	1.00, 1.00	.386
P	1.00	1.00, 1.00	.008	1.00	1.00, 1.00	.956
Se	1.00	1.00, 1.00	.064	1.00	1.00, 1.00	.850
Zin	0.97	0.95, 1.00	.021	0.97	0.93, 1.02	.222
Na	1.00	1.00, 1.00	.041	1.00	1.00, 1.00	.858
K	1.00	1.00, 1.00	.278	1.00	1.00, 1.00	.894
VB1	0.85	0.72, 1.01	.060	0.86	0.63, 1.16	.296
VB12	1.01	0.99, 1.02	.353	1.02	0.98, 1.06	.352
VB2	0.92	0.81, 1.06	.238	1.01	0.81, 1.26	.930
VB6	1.04	0.95, 1.14	.463	1.11	1.00, 1.23	.066
VC	1.00	1.00, 1.00	.339	1.00	1.00, 1.00	.613
VD	0.98	0.95, 1.01	.143	0.98	0.95, 1.02	.264
VK	1.00	1.00, 1.00	.126	1.00	1.00, 1.00	.459

CI = confidence interval, OR = odds ratio, PID = pelvic inflammatory disease.

## 4. Discussion

This cross-sectional analysis, utilizing a robust NHANES cohort, indicated that a higher dietary iron intake correlates with a lower probability of developing PID. Specifically, participants in the highest tertile (T3, 63.1–94.6 mg/day) experienced a 30% reduction in PID risk (T1, < 31.5 mg/day) (*P* = .0231), following adjustments for potential confounders. Additionally, the threshold analysis revealed that each 1-unit increase in dietary iron intake below 27 mg/day led to a 3% decrease in the PID-adjusted ORs (95% CI: 0.95–0.99), with critical points at 27 and 54 mg/day. Notably, substantial negative associations were also detected in subgroups with diabetes and those who do not smoke (*P* < .05). These results advocate that augmenting daily iron intake might be an effective measure to lessen the prevalence of PID.

Contemporary research has increasingly emphasized the influence of dietary factors in obstetrics and gynecology. For instance, the Mediterranean diet has been shown to alleviate pain associated with endometriosis,^[21]^ increased dietary calcium intake has been linked to a reduced risk of ovarian cancer,^[22]^ and the consumption of fruits, vegetables, and green tea offers long-term benefits for gynecological health, whereas fats, red meats, alcohol, and coffee may accelerate the development of such disorders.^[23]^ While PID, a condition characterized by chronic and recurrent inflammatory responses, has been extensively studied, specific recommendations concerning dietary minerals for women with PID remain scarce.

Our supplementary analysis of other dietary trace elements and vitamins further confirmed the specific protective effect of iron on PID: although several elements (Ca, Mg, P, Zn, Na) showed significant correlations with PID in univariate analysis, these correlations disappeared after full covariate adjustment, and no other element presented a significant independent association with PID risk. The marginal protective trend of Zn needs to be verified in larger sample studies or prospective cohort studies, and its potential synergistic effect with iron in PID prevention deserves further exploration.

Iron, a critical trace element, plays an essential role in maintaining overall health, with the majority found in hemoglobin and the rest stored in the liver, myoglobin, and enzymes in a normal physiological state.^[24]^ Plasma iron is mainly derived from iron removed by macrophages from senescent erythrocytes, and some iron ingested from food is reduced from trivalent (Fe3^+^) to divalent (Fe2^+^) by ferric ion reductase, and then absorbed from the intestinal epithelium via divalent metal transporter protein 1.^[25]^The absorption rate of iron in a typical diet is approximately 10 to 15%. However, in cases of iron deficiency within the body, this rate can increase by 2 to 3 times. Other dietary components (such as phytic acid, polyphenols, plant proteins, and certain minerals [e.g., calcium and zinc]) may influence the efficiency of nonheme iron absorption.^[26]^

Previous research has highlighted a modest link between dietary iron and nonpregnant gynecological conditions.^[27]^Such views may be limited. Iron’s crucial role in hemoglobin synthesis often associates it with anemia. Iron-deficiency anemia represents the most common anemia within obstetrics and gynecology.^[28]^ Increased iron demand during pregnancy, due to expanded blood volume and red blood cell production needed for fetal and placental growth, culminating in iron-deficiency anemia. This condition heightens the risk of several complications, including heart disease in pregnancy, puerperal infections, preterm labor, and low birth weight.^[29]^ Moreover, a study has shown that total iron intake from food and supplements early in pregnancy positively correlates with birth weight.^[30]^ Appropriate increase in iron intake improves neurological development in offspring.^[31]^ In nonpregnant women, gynecological conditions such as abnormal uterine bleeding, uterine fibroids, and uterine adenomyosis may lead to excessive menstruation and consequent secondary iron loss, thus depleting iron stores in women of childbearing age,^[24]^ and subsequently increasing the risk of iron deficiency. It has been proposed that iron deficiency-induced hypoxia could disrupt the activity and functionality of bone cells, specifically osteoblasts and osteoclasts, and might negatively affect the synthesis of collagen and vitamin D, possibly leading to bone loss or osteoporosis.^[32]^ In addition to benign diseases, malignant diseases can also lead to conditions such as reduced red blood cell counts or anemia, resulting in iron homeostasis disturbances.^[33]^ Cancer cells increase metabolically available iron not only by increasing iron absorption and reducing its storage, but also by weakening its physiological function.^[34]^ Therefore, most cancer patients suffer from functional iron deficiency.As early as 2010, the guidelines of the European Society for Medical Oncology recommended that all patients should undergo regular monitoring of iron homeostasis (including iron, C-reactive protein, transferrin, and ferritin).

In contrast, iron overload due to excessive dietary iron can induce metabolic dysfunctions and health issues, including heightened risks of coronary heart disease^[35]^ and the development of insulin resistance and type II diabetes.^[36]^ Recent investigations have delved into iron-induced cell death, a distinct type of programmed cell death separate from autophagy, apoptosis, and necrosis, defined by iron overload and lipid peroxidation.^[37]^ Particularly concerning women’s reproductive health, research has shown that excessive supplemental iron intake, specifically above 45 mg/day, might reduce ovarian reserve function,^[38]^ with potential implications for premature ovarian failure. Granulosa cell iron death and oocyte dysplasia were also observed in iron-overloaded follicular fluid, indicating that iron overload can increase the risk of endometriosis-related infertility.^[39]^ Increased iron death was similarly present in the ovarian tissue of patients with polycystic ovary syndrome.^[40]^ Similar mechanisms exist for ovarian cancer; preeclampsia and spontaneous abortion.^[41]^ Additionally, iron overload is recognized as a risk factor for infection, markedly increasing morbidity and mortality in individuals with high iron levels.^[42]^ Iron acts both as a modulator of immune responses and a nutrient for pathogens in infectious diseases, including PID. Study noted that when infection occurs, the host can drive iron withdrawal (sequestering iron in storage compartments, including macrophages) to inhibit pathogen growth which is “trophic immunity.”^[43]^ However, as the infection continues to develop and chronic immunity is activated, it can lead to the isolation of iron from infectious agents; autoreactive lymphocytes; and also from erythroid progenitor cells,^[44]^ which is one of the key mechanisms that lead to anemia in chronic inflammation.

Back to the study of PID in women. The homeostasis of iron as a regulator of the immune system inevitably affects the immune system (innate and adaptive immunity).^[45]^ Innate immunity is the body’s primary defense against pathogens, functioning as a first line of defense through a natural mechanical barrier. Neutrophils, the most significant cells in the innate immune system, possess the capacity to elicit potent antimicrobial effects through the process of Fe3^+^/Fe2^+^ redox-dependent Fe3^+^/Fe2^+^ oxidation by the iron-dependent metalloprotein myeloperoxidase.^[46]^ When pathogens invade, adaptive immunity is rapidly activated.^[47]^ Iron acts as an initiator of adaptive immunity in the clonal expansion of lymphocyte subpopulations.^[48]^ It has been identified as an absorptive material in T cell immunization for timely activation of TfR1 (CD71).^[49]^ In addition to lowering immunity, iron deficiency causes the body to produce more inflammatory factors such as tumor necrosis factor-alpha and interleukin-6.^[50]^ These inflammatory mediators play a key role in the development and progression of PID; they promote infiltration of inflammatory cells and tissue damage, making the inflammatory response more intense.In addition to injury, recent findings indicate that impaired iron homeostasis may contribute to a variety of healing and regenerative processes by inducing iron death.^[51]^ An in vitro study demonstrated that iron promotes differentiation while biasing macrophages to secrete more M2-like polarized states and produce high levels of chemokine (C-C motifs) ligands 17 and 22 promote wound reepithelialization and extracellular matrix deposition in a human model of ex vivo wound healing,^[52]^ which proved iron can aid in the repair and regeneration of pelvic tissue after inflammation, promoting recovery. However, excessive iron intake can also interfere with normal tissue repair processes, increase excessive fibrous tissue proliferation, and other such effects, leading to poor repair and, ultimately, affecting organ function. So it is imperative to ascertain an appropriate range of intake, as this is of the utmost importance.

In summary, the present study lends support to a moderate increase in dietary iron as a protective approach against PID, carefully weighed against the risks of excessive iron intake. However, the limitations inherent to this cross-sectional study must be acknowledged, including the inability to establish causality and potential information biases from questionnaires. As previously mentioned, GI disorders have been shown to result in impaired iron absorption. However, owing to insufficient data in this regard, we were not feasible to incorporate GI disorders as confounders in the study. Furthermore, it is known to us all that sexual activity and the use of sex hormone-based medications can directly influence the development of PID, but given the discrepancy in the available data from the NHANES, we decided that this phenomenon be examined as an exposure factor in a separate study and call for further validation through prospective cohort studies. Future research should focus on identifying precise inflection points for dietary iron that optimize PID prevention and management. Despite dependence on NHANES data potentially limiting the generalizability of our findings, subsequent research will extend existing cohort studies to confirm the relationship between dietary iron intake and PID.

## Acknowledgments

The authors thank all NHANES participants, staff, and investigators. We thank Chen XiaoTeng(Nanjing Medical University) for his experience on the NHANES database. His outstanding work, nhanesR package and installer, makes it easier for us to explore NHANES database.

## Author contributions

**Conceptualization:** Xiaoshi Wang, Jia Ye, Xiaoteng Chen.

**Data curation:** Xiaoshi Wang, Qingsong Zhang, Jinwei Zhang.

**Formal analysis:** Xiaoshi Wang, Qingsong Zhang.

**Funding acquisition:** Jia Ye, Jinwei Zhang.

**Investigation:** Xiaoshi Wang, Qingsong Zhang, Jinwei Zhang.

**Methodology:** Xiaoteng Chen.

**Project administration:** Xiaoshi Wang, Jia Ye, Qingsong Zhang, Jinwei Zhang.

**Resources:** Jia Ye, Qingsong Zhang, Jinwei Zhang.

**Software:** Xiaoshi Wang, Xiaoteng Chen.

**Supervision:** Jia Ye, Xiaoteng Chen, Jinwei Zhang.

**Validation:** Xiaoshi Wang.

**Visualization:** Xiaoteng Chen.

**Writing – original draft:** Xiaoshi Wang, Xiaoteng Chen, Qingsong Zhang.

**Writing – review & editing:** Xiaoshi Wang, Jia Ye, Jinwei Zhang.

## References

[1] Brunham RC Gottlieb SL Paavonen J. Pelvic inflammatory disease. N Engl J Med. 2015;372:2039–48. 25992748 10.1056/NEJMra1411426

[2] Curry A Williams T Penny ML. Pelvic inflammatory disease: diagnosis, management, and prevention. Am Fam Physician. 2019;100:357–64. 31524362

[3] Kaambo E Africa C Chambuso R Passmore JS. Vaginal microbiomes associated with aerobic vaginitis and bacterial vaginosis. Front Public Health. 2018;6:78. 29632854 10.3389/fpubh.2018.00078 PMC5879096

[4] Greydanus DE Cabral MD Patel DR. Pelvic inflammatory disease in the adolescent and young adult: an update. Dis Mon. 2022;68:101287. 34521505 10.1016/j.disamonth.2021.101287

[5] Trent M Ellen JM Frick KD. Estimating the direct costs of pelvic inflammatory disease in adolescents: a within-system analysis. Sex Transm Dis. 2011;38:326–8. 21057380 10.1097/OLQ.0b013e3181fc6c65 PMC4433714

[6] Tako E. Dietary trace minerals. Nutrients. 2019;11:2823. 31752257 10.3390/nu11112823 PMC6893782

[7] Dlouhy AC Outten CE. The Iron metallome in eukaryotic organisms. In: Metallomics and the cell. vol. 12. Banci L, editor. Dordrecht: Springer Netherlands; 2013. p. 241–278. 10.1007/978-94-007-5561-1_8 PMC3924584 23595675

[8] Liu Y Li G Lu F. Excess iron intake induced liver injury: the role of gut-liver axis and therapeutic potential. Biomed Pharmacother. 2023;168:115728. 37864900 10.1016/j.biopha.2023.115728

[9] Bao W Rong Y Rong S Liu L. Dietary iron intake, body iron stores, and the risk of type 2 diabetes: a systematic review and meta-analysis. BMC Med. 2012;10:119. 23046549 10.1186/1741-7015-10-119 PMC3520769

[10] Huang T Cao R Liu P Liu J Yu X. The severity of depression is associated with pelvic inflammatory diseases: a cross-sectional study of the United States national health and nutrition examinations from 2013 to 2018. Front Med (Lausanne). 2022;9:926351. 36314030 10.3389/fmed.2022.926351 PMC9596754

[11] Wu Z Ruan Z Liang G Wang X Wu J Wang B. Association between dietary magnesium intake and peripheral arterial disease: results from NHANES 1999-2004. PLoS One. 2023;18:e0289973. 37566622 10.1371/journal.pone.0289973 PMC10420347

[12] 2015-2020 Dietary guidelines | odphp.health.gov. https://odphp.health.gov/sites/default/files/2019-09/2015-2020_Dietary_Guidelines.pdf. Accessed October 28, 2024.

[13] Fan Y Ni S Zhang H. Associations of copper intake with bone mineral density and osteoporosis in adults: data from the national health and nutrition examination survey. Biol Trace Elem Res. 2022;200:2062–8. 34283365 10.1007/s12011-021-02845-5

[14] Association between dietary magnesium intake and gallstones: the mediating role of atherogenic index of plasma - PubMed. http://pubmed-ncbi-nlm-nih-gov-s.webvpn.njmu.edu.cn:8118/38509591/. Accessed March 20, 2024.

[15] Fan Y Zhang C Bu J. Relationship between selected serum metallic elements and obesity in children and adolescent in the U.S. Nutrients. 2017;9:104. 28165362 10.3390/nu9020104 PMC5331535

[16] Liu H Wang D Wu F Dong Z Yu S. Association between inflammatory potential of diet and self-reported severe headache or migraine: a cross-sectional study of the national health and nutrition examination survey. Nutrition. 2023;113:112098. 37356381 10.1016/j.nut.2023.112098

[17] Maclean RR Cowan A Vernarelli JA. More to gain: dietary energy density is related to smoking status in US adults. BMC Public Health. 2018;18:365. 29614996 10.1186/s12889-018-5248-5 PMC5883399

[18] Correction to: 2018. AHA/ACC/AACVPR/AAPA/ABC/ACPM/ADA/AGS/APhA/ASPC/NLA/PCNA guideline on the management of blood cholesterol: a report of the American college of cardiology/American heart association task force on clinical practice guidelines. Circulation. 2023;148:e5. 37579012 10.1161/CIR.0000000000001172

[19] Johnson CL Paulose-Ram R Ogden CL. National health and nutrition examination survey: analytic guidelines, 1999-2010. Vital Health Stat 2. 2013:1–24. 25090154

[20] Patrician PA. Multiple imputation for missing data. Res Nurs Health. 2002;25:76–84. 11807922 10.1002/nur.10015

[21] Cirillo M Argento FR Becatti M Fiorillo C Coccia ME Fatini C. Mediterranean diet and oxidative stress: a relationship with pain perception in endometriosis. Int J Mol Sci. 2023;24:14601. 37834048 10.3390/ijms241914601 PMC10572576

[22] Sonh X Li Z Ji X Zhang D. Calcium intake and the risk of ovarian cancer: a meta-analysis. Nutrients. 2017;9:679. 28665326 10.3390/nu9070679 PMC5537794

[23] Afrin S AlAshqar A El Sabeh M. Diet and nutrition in gynecological disorders: a focus on clinical studies. Nutrients. 2021;13:1747. 34063835 10.3390/nu13061747 PMC8224039

[24] Andrews NC. Disorders of iron metabolism. N Engl J Med. 1999;341:1986–95. 10607817 10.1056/NEJM199912233412607

[25] Yang J Tang Q Zeng Y. Melatonin: potential avenue for treating iron overload disorders. Ageing Res Rev. 2022;81:101717. 35961513 10.1016/j.arr.2022.101717

[26] Frydrych A Krośniak M Jurowski K. The role of chosen essential elements (Zn, Cu, Se, Fe, Mn) in food for special medical purposes (FSMPs) dedicated to oncology patients-critical review: state-of-the-art. Nutrients. 2023;15:1012. 36839370 10.3390/nu15041012 PMC9961387

[27] Zhu G Li Z Tang L. Associations of dietary intakes with gynecological cancers: findings from a cross-sectional study. Nutrients. 2022;14:5026. 36501056 10.3390/nu14235026 PMC9739794

[28] Kanu FA Hamner HC Scanlon KS Sharma AJ. Anemia among pregnant women participating in the special supplemental nutrition program for women, infants, and children - United States, 2008-2018. MMWR Morb Mortal Wkly Rep. 2022;71:813–9. 35737575 10.15585/mmwr.mm7125a1

[29] Goonewardene M Shehata M Hamad A. Anaemia in pregnancy. Best Pract Res Clin Obstet Gynaecol. 2012;26:3–24. 22138002 10.1016/j.bpobgyn.2011.10.010

[30] Alwan NA Greenwood DC Simpson NAB McArdle HJ Godfrey KM Cade JE. Dietary iron intake during early pregnancy and birth outcomes in a cohort of British women. Hum Reprod. 2011;26:911–9. 21303776 10.1093/humrep/der005 PMC3057752

[31] Iglesias L Canals J Arija V. Effects of prenatal iron status on child neurodevelopment and behavior: a systematic review. Crit Rev Food Sci Nutr. 2018;58:1604–14. 28084782 10.1080/10408398.2016.1274285

[32] Yang J Li Q Feng Y Zeng Y. Iron deficiency and iron deficiency anemia: potential risk factors in bone loss. Int J Mol Sci. 2023;24:6891. 37108056 10.3390/ijms24086891 PMC10138976

[33] Torti SV Torti FM. Iron and cancer: more ore to be mined. Nat Rev Cancer. 2013;13:342–55. 23594855 10.1038/nrc3495 PMC4036554

[34] Lee S Eo W Jeon H Park S Chae J. Prognostic significance of host-related biomarkers for survival in patients with advanced non-small cell lung cancer. J. Cancer. 2017;8:2974–83. 28928889 10.7150/jca.20866 PMC5604449

[35] Sempos CT Looker AC. Iron status and the risk of coronary heart disease: an example of the use of nutritional epidemiology in chronic disease research. J Nutr Biochem. 2001;12:170–82. 11257466 10.1016/s0955-2863(00)00153-4

[36] Jahng JWS Alsaadi RM Palanivel R. Iron overload inhibits late stage autophagic flux leading to insulin resistance. EMBO Rep. 2019;20:e47911. 31441223 10.15252/embr.201947911 PMC6776927

[37] Jiang X Stockwell BR Conrad M. Ferroptosis: mechanisms, biology and role in disease. Nat Rev Mol Cell Biol. 2021;22:266–82. 33495651 10.1038/s41580-020-00324-8 PMC8142022

[38] Jiménez-Cardozo N Mitsunami M Mínguez-Alarcón L. Iron intake in relation to ovarian reserve among women seeking infertility treatment. Hum Reprod. 2023;38:1613–20. 37329261 10.1093/humrep/dead118 PMC10391310

[39] Ni Z Li Y Song D. Iron-overloaded follicular fluid increases the risk of endometriosis-related infertility by triggering granulosa cell ferroptosis and oocyte dysmaturity. Cell Death Dis. 2022;13:579. 35787614 10.1038/s41419-022-05037-8 PMC9253011

[40] Li X Lin Y Cheng X. Ovarian ferroptosis induced by androgen is involved in pathogenesis of PCOS. Hum Reprod Open. 2024;2024:hoae013. 38550897 10.1093/hropen/hoae013 PMC10973940

[41] Liu M Wu K Wu Y. The emerging role of ferroptosis in female reproductive disorders. Biomed Pharmacother. 2023;166:115415. 37660655 10.1016/j.biopha.2023.115415

[42] Vento S Cainelli F Cesario F. Infections and thalassaemia. Lancet Infect Dis. 2006;6:226–33. 16554247 10.1016/S1473-3099(06)70437-6

[43] Nairz M Dichtl S Schroll A. Iron and innate antimicrobial immunity-depriving the pathogen, defending the host. J Trace Elem Med Biol. 2018;48:118–33. 29773170 10.1016/j.jtemb.2018.03.007

[44] Nairz M Weiss G. Iron in infection and immunity. Mol Aspects Med. 2020;75:100864. 32461004 10.1016/j.mam.2020.100864

[45] Nakamura T Naguro I Ichijo H. Iron homeostasis and iron-regulated ROS in cell death, senescence and human diseases. Biochim Biophys Acta Gen Subj. 2019;1863:1398–409. 31229492 10.1016/j.bbagen.2019.06.010

[46] Arnhold J Furtmüller PG Obinger C. Redox properties of myeloperoxidase. Redox Rep. 2003;8:179–86. 14599340 10.1179/135100003225002664

[47] Eisenbarth SC Flavell RA. Innate instruction of adaptive immunity revisited: the inflammasome. EMBO Mol Med. 2009;1:92–8. 20049709 10.1002/emmm.200900014 PMC3378119

[48] Ganz T Nemeth E. Iron homeostasis in host defence and inflammation. Nat Rev Immunol. 2015;15:500–10. 26160612 10.1038/nri3863 PMC4801113

[49] Frost JN Tan TK Abbas M. Hepcidin-mediated hypoferremia disrupts immune responses to vaccination and infection. Med. 2021; 2:164–79.e12. 33665641 10.1016/j.medj.2020.10.004 PMC7895906

[50] Mu Q Chen L Gao X. The role of iron homeostasis in remodeling immune function and regulating inflammatory disease. Sci Bull (Beijing). 2021;66:1806–16. 36654387 10.1016/j.scib.2021.02.010

[51] Yin J Xu X Guo Y. Repair and regeneration: ferroptosis in the process of remodeling and fibrosis in impaired organs. Cell Death Discov. 2024;10:424. 39358326 10.1038/s41420-024-02181-2 PMC11447141

[52] Wilkinson HN Roberts ER Stafford AR. Tissue Iron promotes wound repair via M2 macrophage polarization and the chemokine (C-C Motif) ligands 17 and 22. Am J Pathol. 2019;189:2196–208. 31465751 10.1016/j.ajpath.2019.07.015 PMC12179499

